# Novel species of *Triatoma* (Hemiptera: Reduviidae) identified in a case of vectorial transmission of Chagas disease in northern Belize

**DOI:** 10.1038/s41598-023-50109-0

**Published:** 2024-01-16

**Authors:** Sarah M. Gunter, Alisa Nelson, Alexander R. Kneubehl, Silvia A. Justi, Russell Manzanero, Emily Zielinski-Gutierrez, Claudia Herrera, Julie Thompson, Rajendra Mandage, Hans Desale, Adrianna Maliga, Kim Bautista, Shannon E. Ronca, Francis Morey, Rafael Chacon Fuentes, Beatriz Lopez, Eric Dumonteil, Gerhaldine H. Morazan, Kristy O. Murray

**Affiliations:** 1 Division of Tropical Medicine, Department of Pediatrics, Baylor College of Medicine, Texas Children’s Hospital, Houston, TX USA; 2 William T. Shearer Center for Human Immunobiology, Texas Children’s Hospital, Houston, TX USA; 3 National School of Tropical Medicine, Baylor College of Medicine, Houston, TX USA; 4 Walter Reed Biosystematics Unit, Smithsonian Institution, Museum Support Center, Suitland, MD USA; 5 One Health Branch, Walter Reed Army Institute of Research, Silver Spring, MD USA; 6 Smithsonian Institution National Museum of Natural History, Washington, DC USA; 7 Belize Ministry of Health and Wellness, Belmopan, Belize; 8 Centers for Disease Control and Prevention-Central America Region, Guatemala City, Guatemala; 9 Department of Tropical Medicine, Vector-Borne and Infectious Disease Research Center, School of Public Health and Tropical Medicine, Tulane University, New Orleans, LA USA; 10 Department of Biomedical Science, School of Medicine, Tulane University, New Orleans, LA USA

**Keywords:** Parasitic infection, Entomology

## Abstract

Chagas disease is a leading cause of non-ischemic cardiomyopathy in endemic regions of Central and South America. In Belize, *Triatoma dimidiata* sensu lato has been identified as the predominate taxon but vectorial transmission of Chagas disease is considered to be rare in the country. We recently identified an acute case of vector-borne Chagas disease in the northern region of Belize. Here we present a subsequent investigation of triatomines collected around the case-patient’s home. We identified yet undescribed species, closely related to *Triatoma huehuetenanguensis* vector by molecular systematics methods occurring in the peridomestic environment. The identification of a *T. cruzi*-positive, novel species of *Triatoma* in Belize indicates an increased risk of transmission to humans in the region and warrants expanded surveillance and further investigation.

## Introduction

Chagas disease, caused by the parasite *Trypanosoma cruzi*, is a leading cause of non-ischemic cardiomyopathy in endemic regions of Central and South America^1^. The parasite is predominately transmitted by vectors belonging to the insect subfamily Triatominae (Hemiptera: Reduviidae), commonly referred to as kissing bugs or conenose bugs. The Triatominae subfamily is comprised of over 150 different described species within 18 genera^2,3^. While all species within the Triatominae subfamily are thought to be potential vectors of the parasite, the genus *Triatoma* is one of the most epidemiologically relevant and diverse^2^.

In most of Central America, the currently predominate vector taxon of human health importance is *Triatoma dimidiata* sensu lato (Latreille, 1811)^4^*.* This is a large geographically diverse species complex, with closely related yet distinct species naturally occurring from southern Mexico to northern Peru. The diversity in morphology and ecology throughout the complex’s distribution has led to disagreement over the taxonomy of the group. Most notably, Lent and Wygodzinsky (1979) studied 160 *T. dimidiata* s.l. specimens, concluding that the observed clinal variation was not suggestive of more than one species^5^. More recently, studies have observed significant genetic diversity in the *T. dimidiata* s.l. complex, but the extent and importance of this diversity is still debated^4,6–8^.

In the past four years, molecular and morphological taxonomy made it possible to describe two new species, closely related to *T. dimidiata* s.l.^9,10^. Specifically, in Belize *Triatoma mopan* was identified as a cave-dwelling species in 2018^10^. In Guatemala, *Triatoma huehuetenanguensis* was identified from a collection of *Triatoma* in the department of Huehuetenango^9^. In Yucatan, Mexico, at least two taxa of the *T. dimidiata* complex, including what is now referred to as *T. huehuetenanguensis,* are present in sympatry and frequently hybridize^11^. Additionally, the recent assignment of a neotype for *T. dimidiata*, defining morphologic and molecular characteristics of *T. dimidiata* sensu stricto, was a fundamental start for the understanding of the diversity under the name^8^.

In Belize, *T. dimidiata* s.l. has been identified as the predominant taxon, including *T. huehuetenanguensis* (formerly published as clade III) and hybrids within the complex^12–16^. Based on studies conducted in the country in 2009, domestic infestation was confirmed with prevalent human blood meals in collected vectors^14^. Nevertheless, in 2012, PAHO declared the country free of domestic vector-borne *T. cruzi* transmission, based on lack of detection of human cases during a country-wide seroprevalence study that included >1300 children^17^. In 2018 we began screening patients (adult and pediatric) presenting with acute febrile illness to polyclinics and hospitals across the country for vector-borne diseases, including Chagas disease. Through this effort, we identified an acute case of Chagas disease in a 7-year-old child residing in Sarteneja, Belize^18^. After identifying this case-patient, we conducted vector collection around the case-patient’s home. Here, we describe the molecular identity of the vector species collected and their *T. cruzi* infection status.

## Results

**Figure 1:**
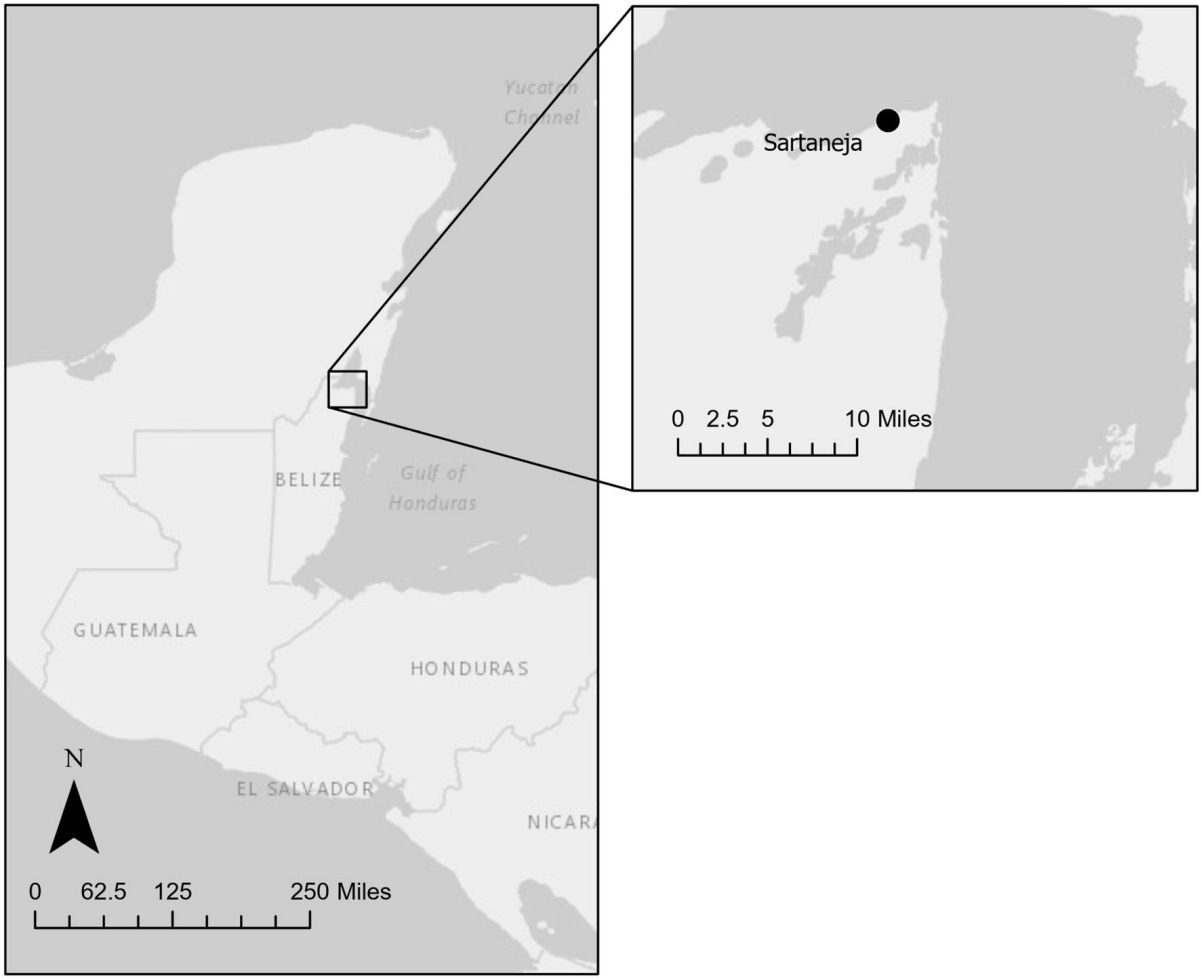
Map of triatomine collection location. Triatomine collections were conducted at the location of the black dot on the map. The map was created using ArcGIS pro V3.0.

**Figure 2:**
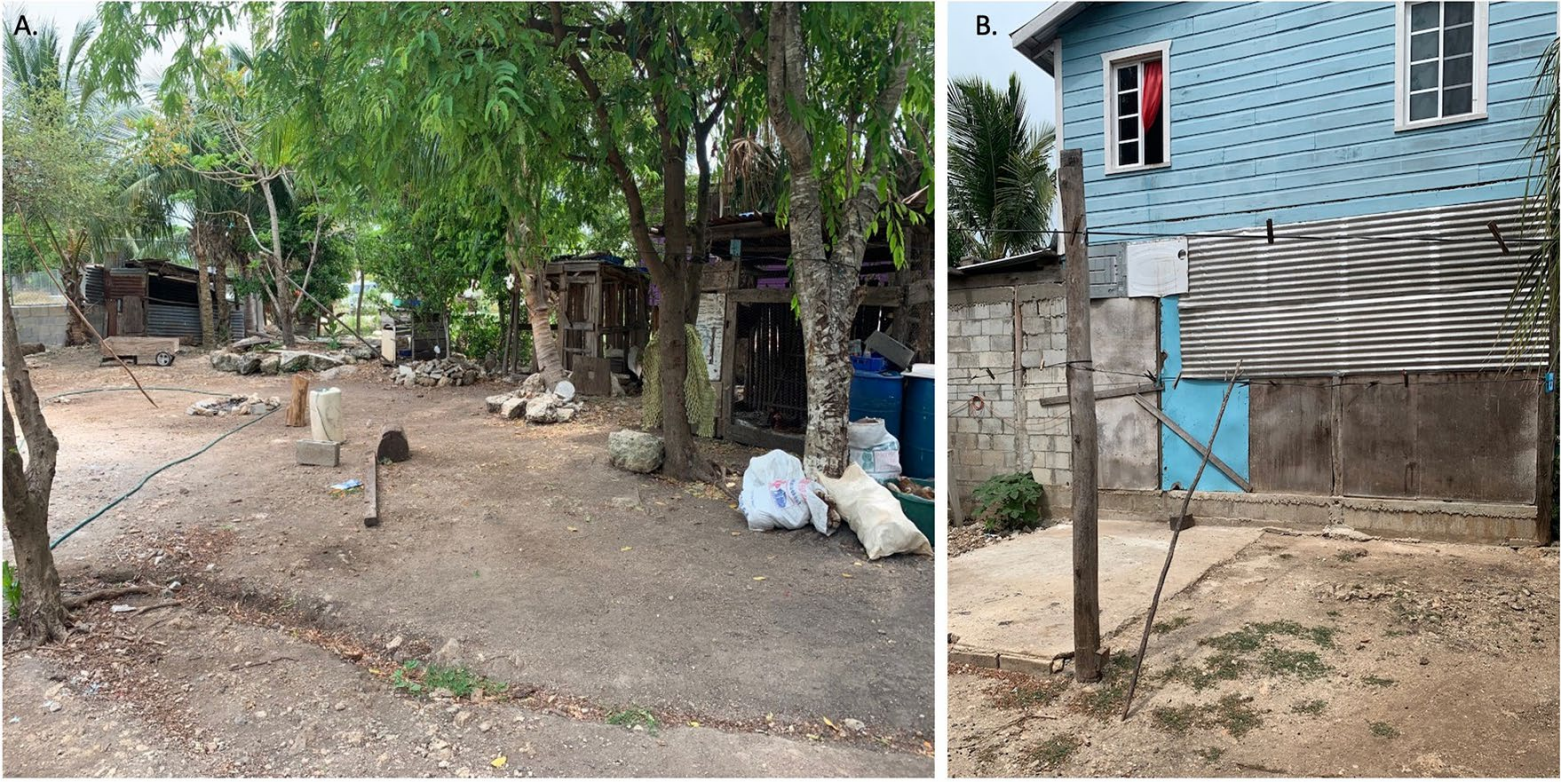
Photographs of case-patient home. Photographs of case-patient home and surroundings were taken during routine triatomine collection. Photograph (**A**) shows the collective animal housing approximately five meters from the home. Photograph (**B**) shows the construction materials used for the home.

The case-patient’s home was located on the periphery of Sarteneja town and was immediately adjacent to a wooded area (Fig. 1). The structure was comprised of cinderblock and tin siding. Additionally, the dwelling was surrounded by wood piles, debris, and collective animal housing. The construction style of the home, adjacent collective animal housing, and proximity to sylvatic environments were recognizable as ideal peridomestic infestation sites for Triatominae species (Fig. 2).

**Figure 3:**
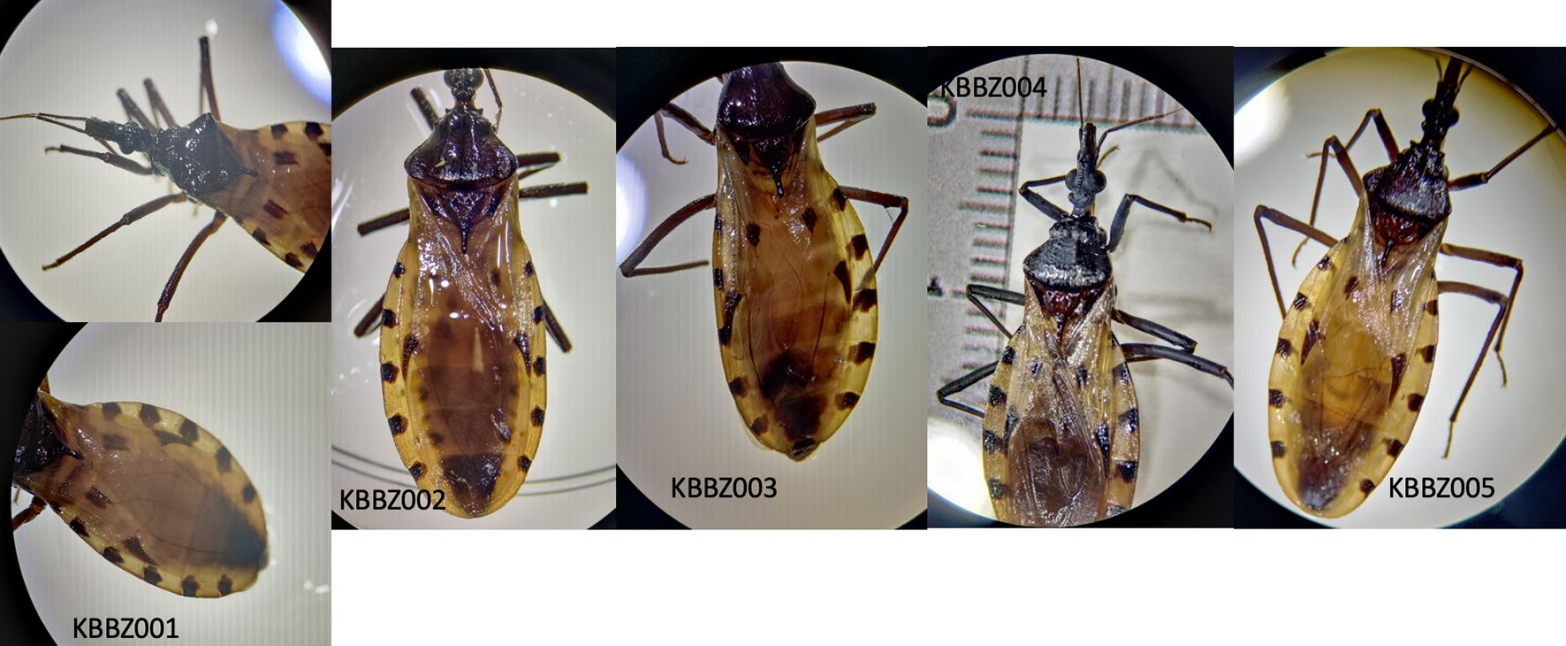
Photographs of collected triatomine samples. Photographs of each specimen collected were taken using a stereoscope with camera adapter. KBBZ001 and KBBZ003-005 are most closely related to *T. huehuetenanguensis* and KBBZ002 is most closely related to *T. dimidiata* s.l.

No *Triatoma* were collected as a result of active collection efforts by the Baylor College of Medicine or Belize Ministry of Health and Wellness teams. However, the family of the case-patient identified five *Triatoma* specimens in and around their house between June 2020 and October 2022 and submitted them for analysis. Initial morphologic examination of the submitted *Triatoma* specimens using the Lent and Wygodzinsky key identified all five as *T. dimidiata,* though differences in size and coloration were noted^5^ (Fig. 3)**.**

**Figure 4:**
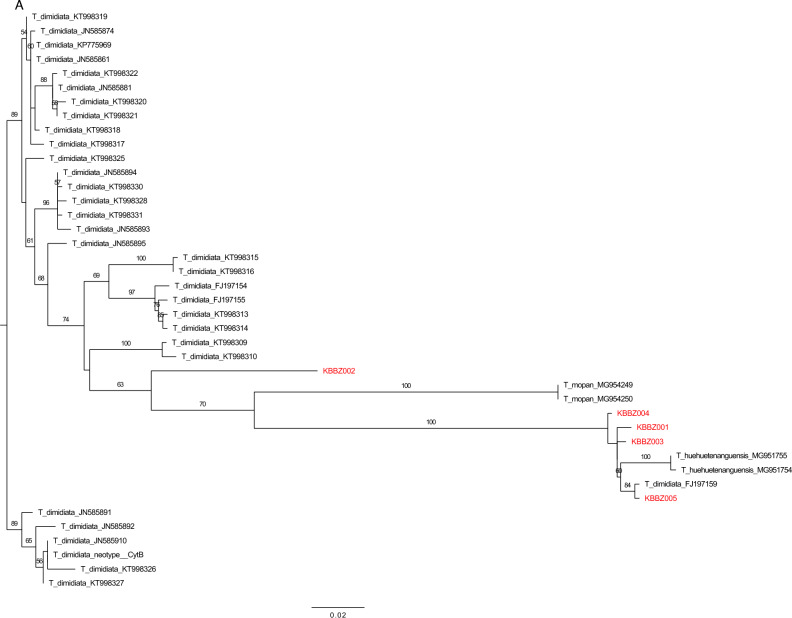

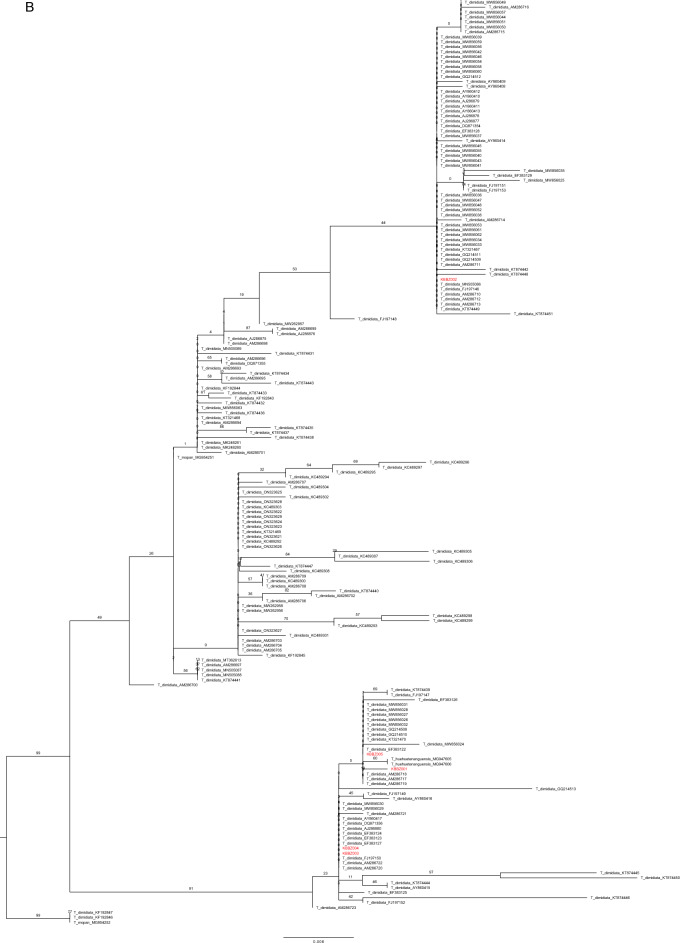
Maximum-likelihood phylogeny of (**A**) CytB and (**B**) ITS-2 sequences. Numbers on clades represent bootstrap support higher than 50. Samples from triatomine collected in this study are in red.

The phylogenetic reconstructions for each marker showed sample KBBZ002 to be closely related to *T. dimidiata* s.l., while the other four were closely related to *T. huehuetenanguensis* (Fig. 4A, B). While samples KBBZ001 and KBBZ003-005 were found to be closely related to *T. huehuetenanguensis,* comparison of the K2P genetic distances between the sequences showed a divergence consistent with interspecific relationship (CytB: mean = 2.57 Std = 0.15; ITS-2: mean = 0.29 Std = 0.15; Table 1), as previously noted^8^. Comparison between KBBZ001 and KBB003-005 sequences were consistent with intraspecific relationships (CytB: mean = 0.91 Std = 0.24; ITS-2: mean = 0.24 Std = 0.15; Table 1)^8^.

**Table 1:** Pairwise K2P distances between the specimens studied and the sequences for ITS-2 and CYTb for the types of *T. huehuetenanguensis*.

	*T. huehuetenanguensis* A10058	*T. huehuetenanguensis* A10227	KBBZ001	KBBZ002	KBBZ003	KBBZ004	KBBZ005
*T. huehuetenanguensis* A10058		0.19	2.58	15.10	2.57	2.77	2.77
*T. huehuetenanguensis* A10227	0.00		2.38	14.84	2.37	2.57	2.57
KBBZ001	0.41	0.49		14.43	0.78	0.98	1.38
KBBZ002	4.01	3.76	4.00		14.50	13.88	14.92
KBBZ003	0.41	0.24	0.41	3.99		0.81	1.14
KBBZ004	0.41	0.24	0.41	3.99	0.00		1.30
KBBZ005	0.20	0.24	0.20	3.78	0.20	0.20	

GenBank accession number for specimens: *T. huehuetenanguensis* A10058 (ITS2-MG947605, CytB-MG951754); *T. huehuetenanguensis* A10227 (ITS2-MG947606, CytB-MG951756). Upper right matrix represents CYTB distances, and lower-left values represent ITS-2 distances.

Mitogenomes were assembled for all collected specimens (Supplemental Fig. 1). All assemblies had the canonical 13 protein coding genes, 22 tRNAs, and 2 rRNAs. Only the KBBZ002 mitogenome was predicted to be circular; however, based on the depth of coverage of the sequence data, all five assemblies were likely incomplete. The read coverage data indicated a substantial increase in read depth within the control region, with unresolved repetitive elements.

Parasite testing data were available for three of the five *Triatoma* samples. Two *Triatoma* specimen closely related to *T. huehuetenanguensis* (KBBZ004-005) did not have the abdomen processed for parasite testing in order to preserve the sample for future morphologic characterization. Our testing identified only one *Triatoma* specimen, belonging to the undescribed group closely related to *T. huehuetenanguensis* (KBBZ003), to be infected with *T. cruzi*.

**Figure 5:**
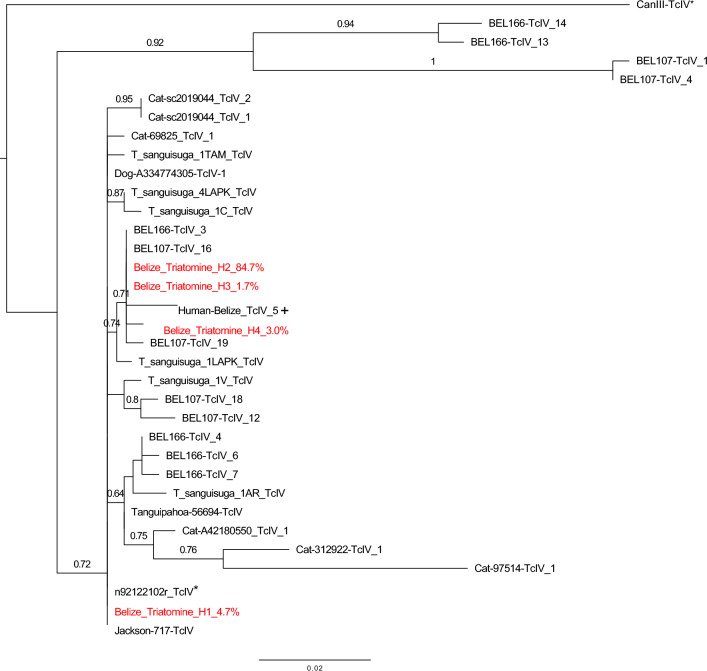
Phylogenetic analysis of TcIV *Trypanosoma cruzi* mini-exon sequences. Mini-exon sequences obtained from the triatomine KBBZ003 (in red) were compared with sequences from reference *T. cruzi* strains (asterisk) and TcIV strains from other triatomine insect vectors, mammals, and the pediatric case of acute Chagas disease (plus sign) using maximum likelihood. Bootstrap support is indicated for the main nodes of the tree for clarity. Percentage values indicated within sequence names refer to the relative proportion of each haplotype identified within the triatomine.

The genotyping of parasite DNA from KBBZ003 resulted in the detection of *T. cruzi* TcIV discrete typing unit (DTU). After mapping using all the DTUs as reference, a total of 89,746 sequences of good quality mapped only to the TcIV DTU. The phylogenetic analysis indicated four unique TcIV min-exon sequence haplotypes, which were most closely related to the TcIV *T. cruzi* sequences identified from *T. dimidiata* vectors previously sequenced from southern Belize^13^ as well as the acute Chagas disease pediatric case-patient^18^ represented in this study. Yet they were distinct from the *T. cruzi* TcIV reference, CanIII, from Brazil (Fig. 5). Reference strains used in the phylogenetic analysis are detailed in Supplemental Table 1.

## Discussion

In this paper, we show the importance of accurately identifying *Triatoma* vectors through molecular techniques, particularly those collected in domestic and peridomestic environments with known vector-human transmission of the parasite *T. cruzi*. From the five *Triatoma* specimens collected (three tested), one was found positive for *T. cruzi*, with the parasite representing a molecular match with the case-patient. Through our investigation, we found this *Triatoma* vector belongs to a taxon that has not yet been formally described; suggesting that simply stating that *T. dimidiata* s.l. or *T*. *huehuetenanguensis* is the vector is insufficient for the region. Clear observable morphological differences to *T*. *huehuetenanguensis*, coupled with the high dissimilarity between the sequences provides evidence that this is a new species, which our group is now characterizing for formal description.

Identification and characterization of novel species is critical for understanding the risk for Chagas disease transmission in the region. Genetic variance in distinct species can signal differences in vector ecology and their ability to transmit disease to humans. Even within the *T. dimidiata* species complex, there have been varying vector ecology, transmission dynamics, and host preferences identified. This newly identified species represents a further divergence from the species complex and represents an undefined risk of disease transmission and ecologic preferences. Such noted diversity may suggest a benefit to further study of triatomine vector species in Central America.

Historically in Belize, the described *T. dimidiata* s.l. and *T. huehuetenanguensis* (formerly published as clade III *T. dimidiata* s.l.) were thought to be predominately sylvatic in nature, but able to seasonally infest houses, and hybrids have also been identified^12,13,15^. While our findings from this small sampling are not robust enough to draw significant conclusions about vector ecology of this potential new species, we made some observations of note. This vector was found in or around a home, was found positive for the parasite, and was also associated with a confirmed case of acute transmission of disease.

The analysis of parasite sequences from this *Triatoma* specimen indicates the presence of the DTU TcIV, which was also detected in the case-patient in whose home the vectors were collected^18^, is closely related to the TcIV reported in North America and distinct from the TcIV from South America. Furthermore, multiple (four) DTU sequence haplotypes of the parasite were found, indicating a high diversity of the parasite circulating in this area and the novel *Triatoma* vector’s potential involvement in transmission.

This study has some noteworthy limitations. Our small sample size of collected triatomine from one geographic area limits our ability to understand the range, prevalence of pathogen, and their vector ecology. Further study is needed to define the risk of human transmission of Chagas disease from this novel species and its distribution in Belize.

## Conclusion

This study represents the first documented identification of a new species closely related to *T*. *huehuetenanguensis*. Despite previous research in the region that described a lack of domestic infection and vector-human transmission in this area, our analysis found this novel species in a peridomestic setting which was in close proximity to the home of a case-patient diagnosed with acute Chagas disease. The identification of a *T. cruzi* positive, novel species of *Triatoma* in Belize indicates a potential increased risk of transmission to humans in the region and warrants expanded surveillance and further investigation. Local public health officials should consider suggesting preventative actions until risk can be defined.

## Methods

### Sample collection

The Belize Ministry of Health and Wellness vector control division and a team from Baylor College of Medicine conducted an environmental investigation for *Triatoma* around the case-patient’s home at multiple time points between 2020 and 2022. Active collections were conducted around the case-patient’s home three times, twice by the Baylor College of Medicine team (October 2021 and June 2022) and once by the Belize Ministry of Health and Wellness team (May 2020). During collection visits the teams educated the family about how to safely collect the vectors if observed. The triatomine samples collected were shipped to the laboratory at Baylor College of Medicine for processing. This study was reviewed and approved by the Institutional Review Board at Baylor College of Medicine (H-44070) and the Belize Ministry and Health and Wellness.

### Sample processing

All samples were initially identified morphologically under a stereoscope using the Lent and Wygodzinsky 1979 keys^5^. Three of the kissing bugs (KBBZ001-003) had the bottom third of the abdomen removed and two (KBBZ004-005) had a leg removed using a sterile scalpel for DNA extraction. For abdominal samples, the removed portion of the abdomen was incubated overnight at 56ºC in Buffer AL and Proteinase K from the Qiagen Blood and Tissue Kit (Qiagen, Germany). For leg samples, the removed portion was frozen in liquid nitrogen and ground using a pestle. Extraction was then completed using Qiagen Blood and Tissue Kit (Qiagen, Germany) per protocol with a final elution in 90 µl. Extracted DNA from abdominal samples was used for *T. cruzi* testing, including DTU identification. Extracted DNA from both abdominal and leg samples was used for molecular confirmation of species.

### Molecular investigation of triatomine samples

Molecular confirmation of species was conducted using Illumina sequencing and subsequent bioinformatics analyses. Specifically, extracted DNA was sent to SEQCENTER (Pittsburgh, PA, USA) for whole genome sequencing. The DNA library was prepared using the Illumina DNA library prep kit (2 × 151 bp reads) and sequenced on an Illumina NextSeq 2000. A minimum of 1Gbp was sequenced for each sample. Adapter sequences as well as sequence with phred scores lower than 20 (−q 20) were filtered using fastp (v0.20.1). Mitogenome sequences were assembled using the filtered Illumina data and the MitoZ pipeline (v3.4) using the ‘all’, ‘--assembler megahit’, ‘--clade Arthropoda’ –genetic_code 5’ options^19^. The annotations generated by this method were manually corrected in Geneious Prime 2022.1.1 to limit gene feature overlaps.

The ITS-2 sequences were determined by performing a separate megahit (v1.2.9) assembly using default parameters for each samples' read data. The contigs of these assemblies were mapped against ITS-2 sequences either from *T. huehuetenanguensis* (MG947605.1) or *T. dimidiata* (MT556666.1 and AM286710.1) using minimap2 (v2.22-r1101)^28^. The contigs that mapped to these ITS-2 sequences were then blasted against GenBank's non-redundant nucleotide database and the ITS-2 sequence was extracted from the contig based on these alignments. The ITS-2, and CytB sequences were blasted against the GenBank's nt database for identity confirmation. Subsequently, these sequences were aligned to all corresponding available ITS-2 and CytB sequences, listed on GenBank as *T. dimidiata*, *T. huehuetenanguensis* and *T. mopan*.

Pairwise Kimura 2-parameter genetic distances were calculated in R, using the package ape^20,21^. Maximum likelihood phylogenies were reconstructed for each alignment, in order to visualize the evolutionary position of our samples. For this, Geneious version Prime 2021.2.2., and the RaxMl implementation, with GTR model and 1000 bootstrap pseudoreplicates. A summarized distance matrix can be found in Table 1, while the whole matrix and phylogenies are displayed in Fig. 4A, B.

### *Trypanosoma cruzi* infection detection

Samples were tested for the presence of *T. cruzi* using the TcZ1/TcZ2 primer set that targets the pathogens repetitive genomic satellite DNA (satDNA), using PCR conditions described previously^22,23^. The sample found positive by initial screening had the DTU identified. Specifically, parasite genotyping was performed using two PCR primer sets targeting the mini-exon gene sequence, including Souto primers^24^, which give PCR products of different sizes according to the DTU, as well as newly designed primers that amplify a larger fragment of 500 bp of this marker^25,26^. Two reference strains were used as positive controls, WB1 (TcI) and Esmeraldo (TcII), and no template DNA (molecular grade water) was used as a negative control. PCRs were performed using previously reported PCR conditions^24,25^. PCR products were separated on a 2% agarose gel stained with ethidium bromide and purified using a PureLink Quick PCR Purification Kit (Invitrogen, Waltham, MA, US). Amplicons were processed for Nanopore library preparation using the Rapid Barcoding Sequencing (SQK-RBK004) protocol and sequenced using the MinION sequencing platform (Oxford Nanopore Technologies, Oxford, UK) on a R9.4.1 flow cell, and basecalled using MinKNOW with high accuracy setting. Raw reads had an average phred score of 16.2, and only consensus haplotypes of mini-exon sequences representing > 1% of amplicon reads were retained.

### *Trypanosoma cruzi* Phylogenetic analysis

Parasite genotyping read quality was assessed using the FastQC tool v0.11.9 with default parameters^27^. Sequence reads were then separately mapped to *T. cruzi* mini-exon reference sequences representing 7 DTUs using minimap2 with default settings^28^. Manual inspection of aligned reads was done with the Integrative Genomics Viewer (IGV) browser to ensure complete coverage mapping with DTU reference sequences. FreeBayes SNP/variant calling tool was used to distinguish sequence variants from sequence artifacts, as implemented in Geneious Prime 2022.1.1, and only sequences representing ≥ 1% of the total sample were included in further analyses. Muscle alignments were performed with all haplotypes and reference strains from all DTUs to precisely identify DTUs, and separate analyses were performed for the TcIV DTU to improve comparisons of closely related sequences. Phylogenetic trees based on maximum likelihood were constructed using the Phylogeny.fr platform^29^.

### Supplementary Information

Supplementary Table 1.

Supplementary Figure 1.

Supplementary Legends.

## Data Availability

The authors declare that all data supporting the findings of the study are available in this article and its Supplementary files, or from the corresponding authors. Sequencing data generated from the triatomine samples are associated with the BioProject PRJNA874442 and BioSamples SAMN30498312-SAMN30498316. The Illumina sequencing reads generated from the *Triatoma* in the Sequence Read Archive (SRA accession #SRR21389601-SRR21389605). The mitogenome assemblies were deposited in GenBank OP345447-OP345451.
