# Supplementary material for: Novel species of Triatoma (Hemiptera: Reduviidae) identified in a case of vectorial transmission of Chagas disease in northern Belize
Source: Sci Rep. 2024 Jan 16;14:1412. doi: 10.1038/s41598-023-50109-0 (PMC10792162; doi:10.1038/s41598-023-50109-0)
Supplement: Supplementary file 1 — Supplementary Table 1. [file 41598_2023_50109_MOESM1_ESM.pdf]

Supplemental Table 1. ***T. cruzi*** **mini-exon reference sequences.**

DTU	Strain	Genbank Accession Number
Initial mapping		
TcI	H1	EF576846
TcII	Tu18	AY367125
TcIII	M5631	AY367126
TcIV	92122102r	AY367124
TcV	SC43	AY367127
TcVI	CL	U57984
TcBat	TCC2477	KT305884
Additional references		
TcIV	CanIII	AY367123.1
	T_dimidiata_BEL166 H1	MW861768
	T_dimidiata_BEL166 H2	MW861767
	T_dimidiata_BEL166 H3	MW861771
	T_dimidiata_BEL166 H4	MW861772
	T_dimidiata_BEL107 H1	MW861764
	T_dimidiata_BEL107 H2	MW861761
	T_dimidiata_BEL107 H3	MW861765
	T_dimidiata_BEL107 H4	MW861762
	Cat-97455 H1	MW477929
	Cat-sc2019044 H1	MW477927
	Cat-A42180550 H1	MW477924
	Cat-312922 H1	MW477925
	Cat-97514 H1	MW477923
	Dog-A34613162 H1	MT365418
