# Supplementary figures and images for: Novel species of Triatoma (Hemiptera: Reduviidae) identified in a case of vectorial transmission of Chagas disease in northern Belize
Source: Sci Rep. 2024 Jan 16;14:1412. doi: 10.1038/s41598-023-50109-0 (PMC10792162; doi:10.1038/s41598-023-50109-0)

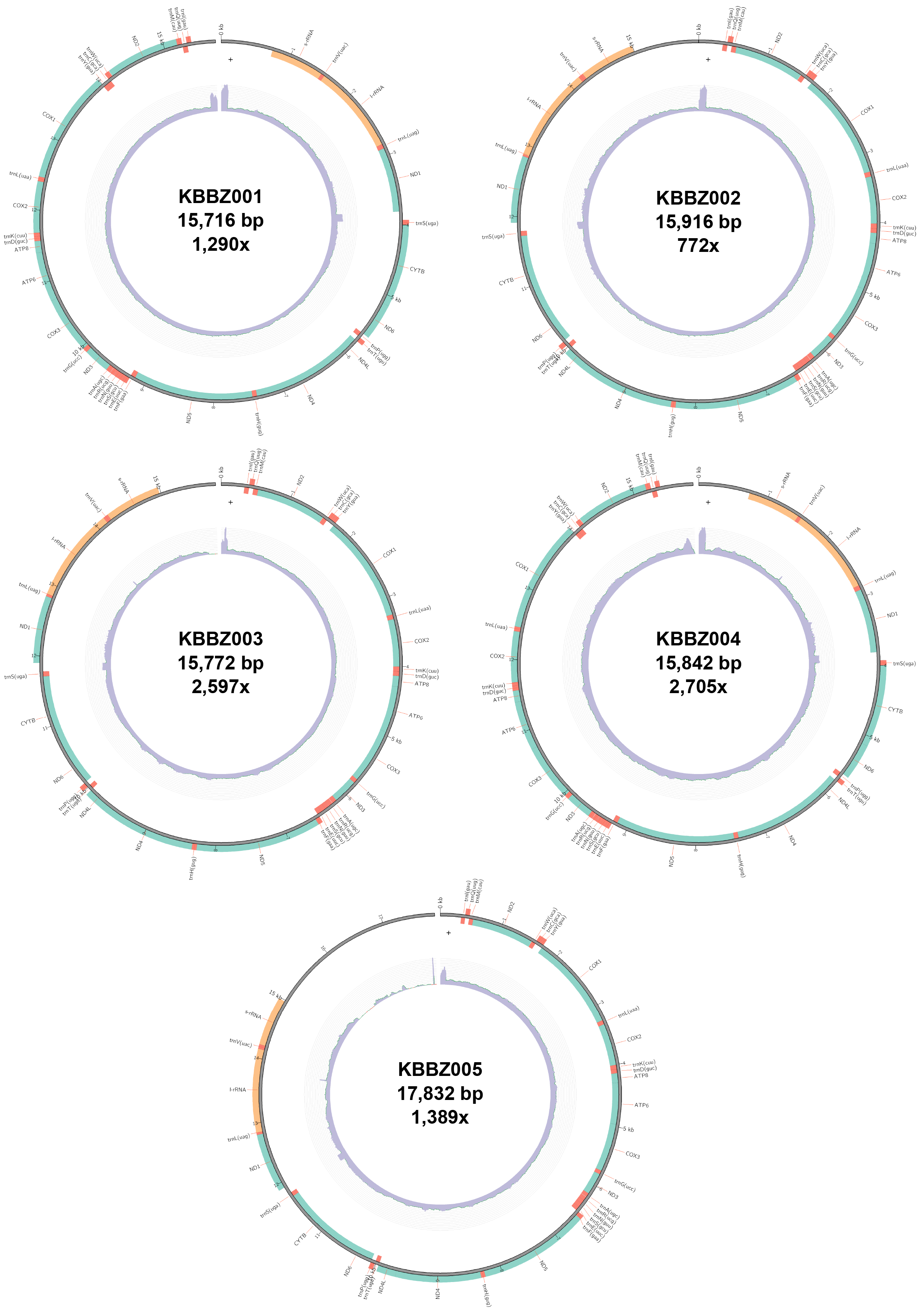

Supplement: Supplementary file 2 — Supplementary Figure 1. [file 41598_2023_50109_MOESM2_ESM.tif]
