# Supplementary material for: Novel species of Triatoma (Hemiptera: Reduviidae) identified in a case of vectorial transmission of Chagas disease in northern Belize
Source: Sci Rep. 2024 Jan 16;14:1412. doi: 10.1038/s41598-023-50109-0 (PMC10792162; doi:10.1038/s41598-023-50109-0)
Supplement: Supplementary file 3 — Supplementary Legends. [file 41598_2023_50109_MOESM3_ESM.docx]

**Supplemental Figure 1- Mitogenome Assembly**

Graphical depiction of assemblies for triatomine samples KBBZ001-005. Circular mitogenome maps were generated by MitoZ. Features found within the outer circle are in the positive sense and feature found outside the outer circle are in the negative sense. Green features indicate protein coding genes, red features indicate tRNAs, and orange features indicate rRNAs. The inner circular graph depicts read coverage across the assembly. The sample, the size in base pairs, and the mean depth of coverage (calculated by mosdepth v0.3.3)(30) are indicated in the center of each mitogenome figure.
